# Effects of germinating temperature and time on metabolite profiles of sunflower (*Helianthus annuus* L.) seed

**DOI:** 10.1002/fsn3.1983

**Published:** 2021-05-05

**Authors:** Shuangshuang Guo, Utai Klinkesorn, Yaowapa Lorjaroenphon, Yan Ge, Kriskamol Na Jom

**Affiliations:** ^1^ Department of Food Science and Technology Faculty of Agro‐Industry Kasetsart University Bangkok Thailand; ^2^ International Hospitality & Dietary Culture College Nanjing Tech University Pujiang Institute Nanjing China; ^3^ Plant Phenomics Research Center Nanjing Agricultural University Nanjing China; ^4^ College of Engineering Nanjing Agricultural University Nanjing China

**Keywords:** germination, metabolite profiles, sunflower seed

## Abstract

Sprouts with higher levels of nutrients and lower content of antinutritional substances have been gained a growing interest in the influence on the human's health. The study of the influence of germination temperature and time on the metabolite profiles of sunflower seed was studied by a metabolomics approach based on gas chromatography–flame ionization detection (GC‐FID). Samples were extracted and fractionated covering a wide range of lipophilic and hydrophilic spectra. A total of 90 metabolites were identified by comparison with reference standards. Principal component analysis (PCA) revealed distinct dynamic changes in metabolites with the germinating time. Heatmap and agglomerative hierarchical clustering analysis revealed the differences and similarities among the samples. The germinating sunflower seeds clustered into three major groups. For instance, group I with a high content of sterols, monosaccharide, and amino acids, indicating the germination process, resulted in an increase in amino acids and monosaccharide. Group II had a high content of FAME and FFA. Relative targeted quantification of metabolites visually depicted by heatmap showed decreases in fatty acid methyl ester (FAME) and free fatty acid (FFA), and increases in amino acids, α‐tocopherol, sterols, and γ‐aminobutyric acid (GABA) during germination. Sunflower seeds germinated at 25°C were better for the accumulation of α‐tocopherol, stigmasterol, leucine, proline, methionine, glutamine, and GABA compared with those at 35°C. These results help to better understand how germination conditions change the nutritional quality of germinated sunflower seeds from a metabolite profile view, allowing for the rational screening and usage of germinated sunflower seeds in the food industry.

## Graphical abstract

1. Germination increased amino acids, α‐tocopherol, sterols, and GABA contents. 2. Germination reduced fatty acids content. 3. Optimal germinating condition was 25°C in the dark

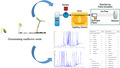

## 1 INTRODUCTION

*Helianthus annuus* L. belongs to the *Asteraceae* family, which is planted commercially all over the world and has many nutritional and medicinal values. It is not only an important source of edible oil, but also a popular snack in the Mediterranean, Eastern European, and Asian countries (Fu et al., 2020). Sunflower seeds are an excellent source of vitamin E and polyunsaturated fatty acids (Cho et al., 2008). These natural antioxidants and polyunsaturated fatty acids have protective effects on hypertension and cardiovascular disease (Cho et al., 2008).

Germination is an important stage of plant development. This stage of the plant life cycle has a combination of multiple catabolic and anabolic processes and also being considered to improve the medicinal qualities of seed (El‐Adawy et al., 2003). Some metabolites change in the seed during germination, such as storage lipids, proteins, carbohydrates, and other seed storage metabolic substances (Satyanarayana et al., 2011). The starch was hydrolyzed to free sugars because of amylase activation during germination (Lorenz, 1980). The protein bioaccessibility and digestibility were found to improve due to the germination remove or repress protease inhibitors (Ohanenye et al., 2020). The lipid content of nongerminated seeds was significantly higher than that of seeds germinated for 48 hr (Xu et al., 2017). Sunflower sprouts have higher antioxidant activity than sunflower seeds, mainly due to the increase of total phenolic, melatonin, and total isoflavone contents during sprouting (Cho et al., 2008).

Seed germination is dependent upon a multitude of endogenous and exogenous factors such as temperature. The temperature is the main factor that regulates the germination process of seed (Vicente et al., 2020). There are three cardinal temperatures that germination response to temperature: base, optimal, and ceiling temperature. The germination rate at base and ceiling temperatures is zero, whereas the highest germination rate is observed at the optimum temperature (Alvarado & Bradford, 2002). However, faster germination does not necessarily mean a better performance during seed germination. Seeds prepare their biochemical machinery and molecular to support successful seedling establishment. The high temperature might have a negative influence on important metabolic processes, such as fatty acid oxidation, storage oil breakdown, and gluconeogenesis(Ribeiro et al., 2015). There is still a lack of information about the correlation between germination and metabolic changes under different temperatures.

Metabolite profiling aims to extract, separate, and analyze as wide a spectrum of metabolites as possible from complex matrices in an efficient and reproducible manner (Shu et al., 2008). The method of metabolite analysis based on capillary gas chromatography (GC) combined with powerful statistical tools has been proved to be a suited platform for the comprehensive study of plant‐derived food. For example, metabolite profiling‐based GC: revealed differences in *Tirgonella foenum‐graecum* species (Farag et al., 2016); was used as an evaluating index for the detection of storage quality and flavor in squash (Okazaki et al., 2016), pea shoots (Santos et al., 2014), and mandarins (Goldenberg et al., 2016); and monitored time‐dependent metabolic changes in plants and crops during their natural development, like potatoes (Davies, 2007), strawberries (Fait et al., 2008), and mung beans(Na Jom et al., 2015).

Therefore, the use of metabolomics is very important for studying the germination process of sunflower seeds. The effects of germination conditions on metabolite profiles of sunflower seed during germination are poorly known. The objective of the study is to analyze the impact of germination conditions on metabolite profiles during the seed germination process. The information gained would provide a better comprehension of the metabolism of sunflower seed germination.

## 2 MATERIALS AND METHODS

### 2.1 Material

Sunflower seeds were harvested in 2017 and purchased from the local market in Thailand. The seeds were collected in plastic bags and stored at 4°C for further analysis.

### 2.2 Reagents

Hydroxyl ammonium chloride, pyridine, methyl tertiary‐butyl ether, and sodium methylate for the extraction and derivatization were HPLC grade. N‐trimethylsilylimidazole and N‐Methyl‐N‐(trimethylsilyl) trifluoroacetamide were GC derivatization grade. Undecane, hexadecane, tetracosane, triacontane, and octatriacontane were retention time standards; Tetracosane, 5α‐cholestan‐3ß‐ol, phenyl‐ß‐D‐glucopyranoside, and *p*‐chloro‐L‐phenylalanine were internal standards; and all reference standards were obtained from Sigma Aldrich Co. (St.Louis, MO, USA). Acetonitrile, hexane, methanol, and dichloromethane were analytical grade purchased from RCI Labscan Ltd. (Pathumwan, Bangkok, Thailand).

### 2.3 Germinating and preparation of sample

Sunflower seeds were germinated in a constant climate chamber with controlled humidity model KBF 720 (Binder, Tuttlingen, Germany) at 25 and 35°C, 75% relative humidity. The samples were taken at 24, 48, 72, 96, and 108 hr, and freeze‐dried in a freeze dryer (GAMMA 1–16 LSC, Martin Christ, Gefriertrocknungsanlagen GmbH, Germany). Dried samples were ground into fine flour and stored until analysis at −20°C in a tightly sealed aluminum bag.

### 2.4 Metabolite profiling

The metabolite profiling procedures of freeze‐dried samples were described as previously procedures by Na Jom et al. (2011) with some modifications. Lipids were transesterified in methanol and then separated into two fractions by solid‐phase extraction (SPE). Fraction I contained—FAME and hydrocarbon, and fraction II contained minor lipids (FFA and sterol). The polar extract was separated into fraction III (silylated sugar and sugar alcohol) and fraction IV (organic acid and amino acid) by selective hydrolysis of silylated derivatives. All fractions were analyzed using HP 6890 model GC‐FID (Agilent Technologies, Palo Alto, CA). The chromatography column used for analysis is a DB‐1, 60 m × 0.32 mm i.d. fused silica capillary coated with a 0.25 μm film of poly‐dimethylsiloxane (Agilent Technologies, California, USA). Helium was at a constant flow rate of 1.8 ml/min. The splitless injection was performed at 280°C. The column temperature was programmed initially at 100°C, increased to 320°C at 4°C/min, and hold for 15‐min. Each sample was tested three times under the same conditions for better repeatability.

### 2.5 Statistical analysis

Chromatographic data were collected and integrated by an HP ChemStation A.06.03. The metabolites were identified by comparison of the retention time with those of the reference standards. PCA and agglomerative hierarchical clustering analysis were used for the classification of the germinating sunflower seeds based on metabolites. Analysis of variance was obtained from a one‐way ANOVA with a 95% significance level (Tukey's range test). All statistical analyses were conducted by XLSTAT (Addinsoft, NY, USA, version 2016.7).

## 3 RESULTS AND DISCUSSION

### 3.1 Capillary gas chromatograms of fraction I‐IV

**FIGURE 1:**
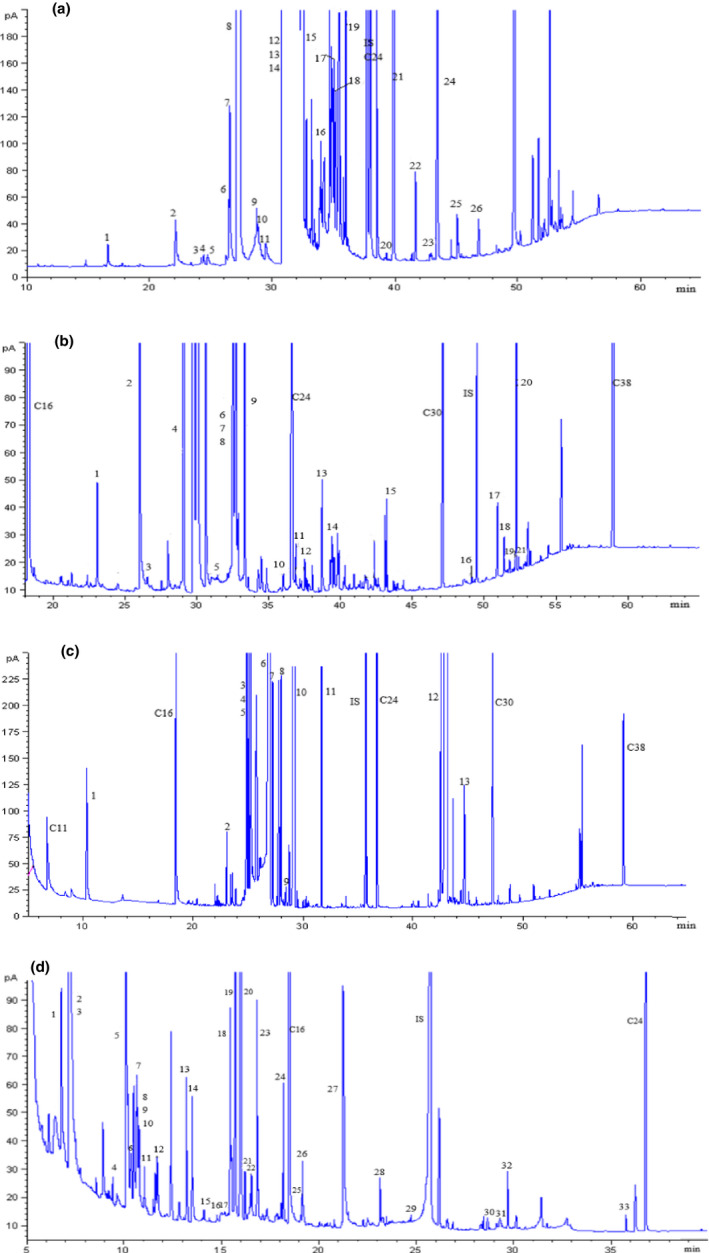
GC/FID chromatograms of fractions I (a), II (b), III (c), and IV (d) from the germinated sunflower seed. Peaks identified were given in Tables 1 and 2

GC‐FID chromatograms of polar and nonpolar fractions obtained from the sunflower seed germinated at 25°C were shown in Figure 1. Fraction I and fraction II were separated by SPE after transesterification. Fraction III and fraction IV were separated after silylation and subsequent differential hydrolysis due to the relative stability of the $R‐\mathrm{Si}{\left({\mathrm{CH}}_{3}\right)}_{3}$ group of sugars/polyols and aqueous hydrolysis of organic acids. A total of 90 metabolites were identified by comparison with reference standards from germinating sunflower seed. These compounds were divided into four groups: fraction I including 23 FAMEs and 5 hydrocarbons; fraction II including 9 FFA, 4 fatty alcohols, 5 sterols, and α‐tocopherol; fraction III including 10 sugars and sugar alcohols; and fraction IV including 25 amino acids and 8 organic acids (Tables 1 and 2).

**TABLE 1:** Compounds identified in fraction I and fraction II of germinating sunflower seed

No.Compound	Ident.^1^	No.Compound	Ident.	No.Compound	Ident.
Saturated FAME		Unsaturated FAME		Hydrocarbons	
2 C14:0	A	3 C15:1	A	1 C14	A
5 C15:0	A	6 C16:1	A	4 C18	A
8 C16:0	A	7 C16:2	A	10 C20	A
11 C17:0	A	9 C17:1	A	16 C22	A
15 C18:0	A	12 C18:3	A	25 Squalene	A
19 C20:0	A	13 C18:2	A		
21 C22:0	A	14 C18:1	A	Sterols/stanol	
22 C23:0	A	17 C20:2	A	17 Campesterol	A
24 C24:0	A	18 C20:1	A	18 Stigmasterol	A
26 C26:0	A	20 C22:1	A	19 △7‐campesterol	A
		23 C24:1	A	20 β‐Sitosterol	A
Free fatty acids		Fatty alcohols		21 Sitostanol	A
1 C14:0	A	5 C18:0‐OH	A		
3 C15:0	A	10 C20:0‐OH	A	Hydroxy FAME	
4 C16:0	A	14 C22:0‐OH		13 9,12‐OH 18:0	A
6 C18:3	A	15 C24:0‐OH	A		
7 C18:2	A				
8 C18:1	A	16 α‐Tocopherol	A	2 Methyl ferulate	A
9 C18:0	A				
11 C20:1	A				
12 C20:0	A				

**TABLE 2:** Compounds identified in fraction III and fraction IV of germinating sunflower seed

No.Compound	Ident.^2^	No.Compound	Ident.
Sugars and sugar alcohols		4 β‐Alanine	A
1 Glycerol	A	5 Valine	A
2 Ribitol	A	8 Leucine	A
3, 4, 5 Fructose	A	9 Isoleucine	A
6, 10 Glucose	A	10 Proline	A
7 Galactose	A	11 Glycine	A
8 Mannitol	A	14 Serine	A
9 Sorbitol	A	15 Threonine	A
11 Myo‐inositol	A	16 β‐Alanine	A
12 Sucrose	A	18 β‐Aminoisobutyric acid	A
13 Trehalose	A	20 Pyroglutamic acid	A
		21 Methionine	A
		22 Aspartic acid	A
Acids		21 Pyroglutamic acid	A
1 Hdrooxyacetic acid	A	25 Glutamic acid	A
6 4‐Hydroxybutyric acid	A	26 Phenylalanine	A
7 Phosphoric acid	A	27 Asparagine	A
12 Fumaric acid	A	28 2‐Aminoadipic acid	A
13 Pyrole‐carboxylic acid	A	29 Glutamine	A
17 2‐Piperidinecarboxylic acid	A	30 Histidine	A
19 Malic acid	A	31 Lysine	A
24 Threonic acid	A	32 Tyrosine	A
		33 Tryptophan	A
Amino acids		23 GABA	A
2 Alanine	A		
3 Glycine	A		

### 3.2 Multivariate analysis

**FIGURE 2:**
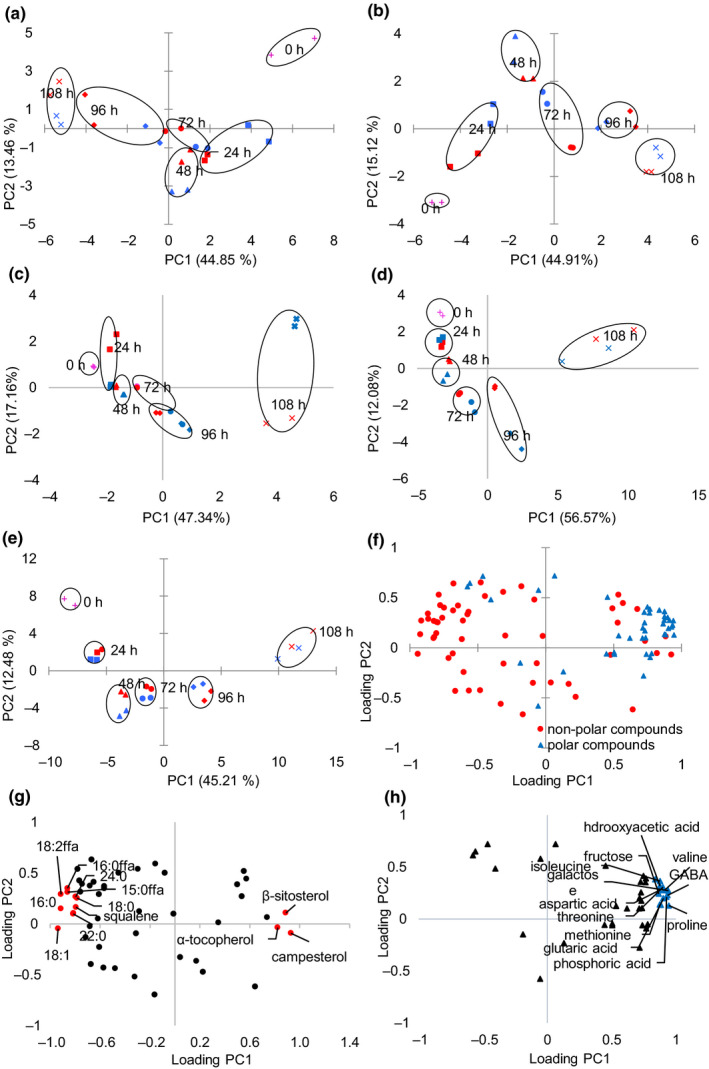
Principal component analysis from all identified metabolites. Score plot of fraction I (a), fraction II (b), fraction III (c), fraction IV (d), and combined fractions I‐IV (e). Blue represents 25°C, and red represents 35°C. Loading plot of nonpolar and polar compounds (fractions I‐IV) (f), nonpolar compounds (fractions I and II) (g), and polar compounds (fractions III and IV) (h). Red circles and blue triangles indicate the compounds with the highest PC1 and PC2 loading scores

Multivariate statistical techniques such as PCA and hierarchical clustering analysis have been used for the grouping of samples based on similarities in metabolic profiles. Heatmap is another way to visive hierarchical clustering where data values are transformed to color scale for the visualization and displaying data matrix. Visualizing the data matrix in this way can help to find the variables that appear to be characteristic for each sample cluster. Based on the data of 90 detected compounds, the PCA score plot and loading plot of the combined and single fraction were shown in Figure 2. The time‐dependent shift of the scores of PC1 and PC2 reflects the changes in metabolism. The two temperatures showed similar score patterns at different germination times. The first two principal components accounted for 56.99% of the total variance based on the analysis data of the metabolite combination of all four fractions (Figure 2e). For the nonpolar fraction, PC1 and PC2 covered 58.31% of the total variation in fraction I (Figure 2a) and 60.03% in fraction II (Figure 2b). PC1 and PC2 of polar fraction explain more significant variance: 64.5% in fraction III (Figure 2c) and 68.65% in fraction IV (Figure 2d), respectively. A similar variance range has been reported in the previous study (Shu et al., 2008). Metabolites in the early germinating times separated (0, 24, 48 hr) from the late germinating times (96 and 108 hr). Sunflower seed samples at 0, 24, 48, and 72 hr were placed on the right side of PC1, and samples at 96 and 108 hr were placed on the left side of PC1 (Figure 2a). While along with PC1 in fractions II, III, and IV, samples at 0, 24, 48, and 72 hr were clustered on the left while samples at 96 and 108 hr were on the right (Figure 2b,c,d). From the loading plot (Figure 2f‐h) along PC1, nonpolar compounds were separated from polar compounds. The results indicated that polar compounds were positively correlated with samples at the late germinating times and nonpolar compounds in fraction I were negatively correlated with samples at the late germinating times. To evaluate the main contributors to the time‐dependent separation of sunflower seed germination, loading scores of the principal components were examined the variation between 25 and 35°C (Figure 2g,h). Higher positive loadings of polar compounds (Figure 2h) along PC1 were composed of fructose, galactose, 3 organic acids (phosphoric acid, glutaric acid, and hydroxyacetic acid), and 7 amino acids (isoleucine, valine, threonine, methionine, proline, aspartic acid, and GABA). Higher negative loadings of nonpolar compounds along PC1 (Figure 2g) were associated with 3 free fatty acids (C15:0, C16:0, and C18:2), 5 FAMEs (C16:0, C18:0, C18:1, C22:0, and C24:0), and squalene. α‐Tocopherol, β‐sitosterol, and campesterol were positive associated with the germinating time.

**FIGURE 3:**
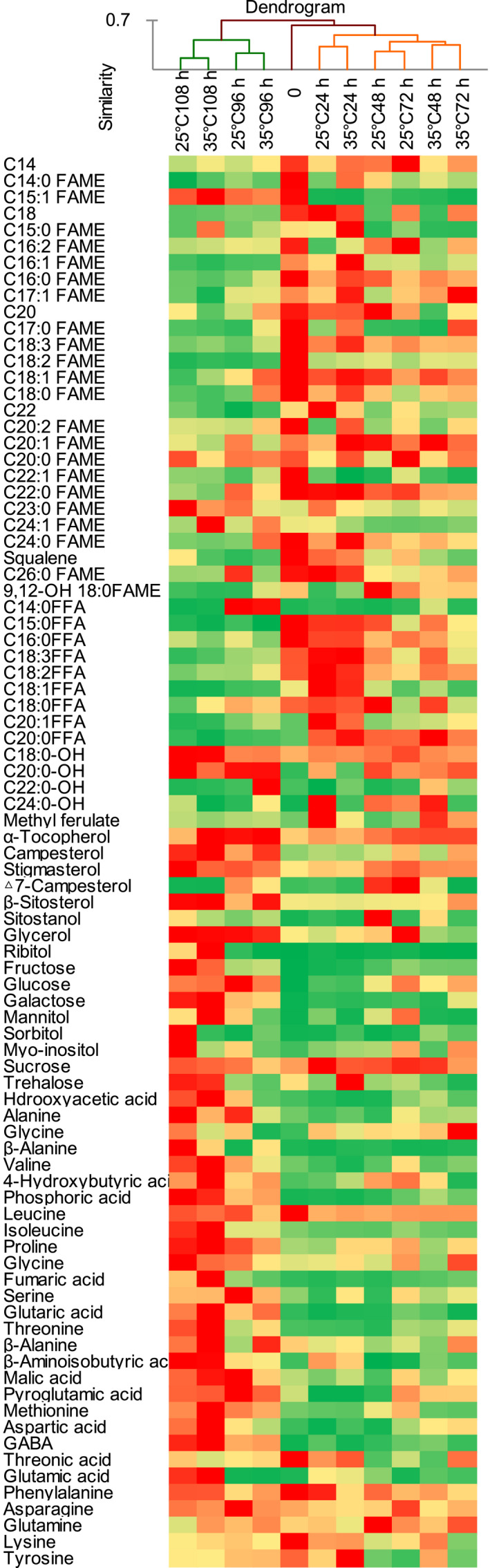
Hierarchical cluster analysis and heatmap showing the coordinated changes of the metabolites during seed germination. The deeper the red color, the higher content; similarly, the deeper the green color, the lower the content

To examine the distribution of the individual metabolites among different germinating temperatures and times, the relative content determined for each compound in 33 samples was scaled and a hierarchical cluster analysis was performed. The results were visualized in a heatmap diagram that was combined with agglomerative hierarchical clustering of the metabolites which were the main compounds identified in germinating sunflower seed (Figure 3). The grouping result of hierarchical agglomerative clustering followed the PCA result with three groups distinguished: without germination (group II), early germination stage group (group III), and late germination stage group (group I). Group I consisted of the samples germinated for 96 and 108 hr at 25 and 35°C with a high content of sterols, monosaccharide, and amino acids, indicating the germination process resulted in an increase in amino acids and monosaccharide. Group II consisted of the sample without germinating, and the sample had a high content of FAME and FFA. Group III consisted of the samples germinated for 24, 48, and 72 weeks at 25 and 35°C.

### 3.3 Relative quantifications

**FIGURE 4:**
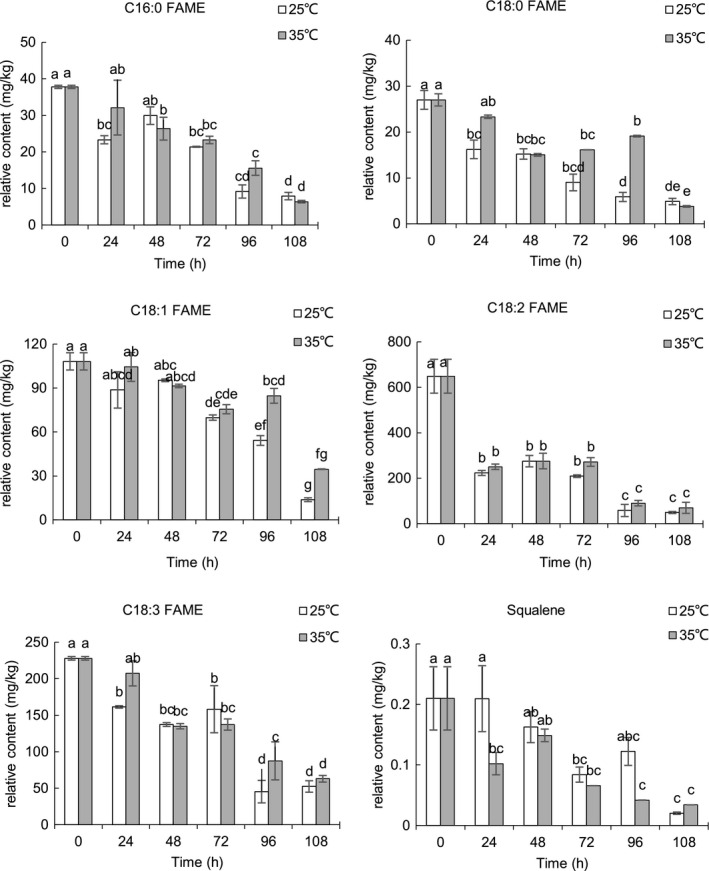
Relative content of representative compounds in fraction I of germinating sunflower seed

**FIGURE 5:**
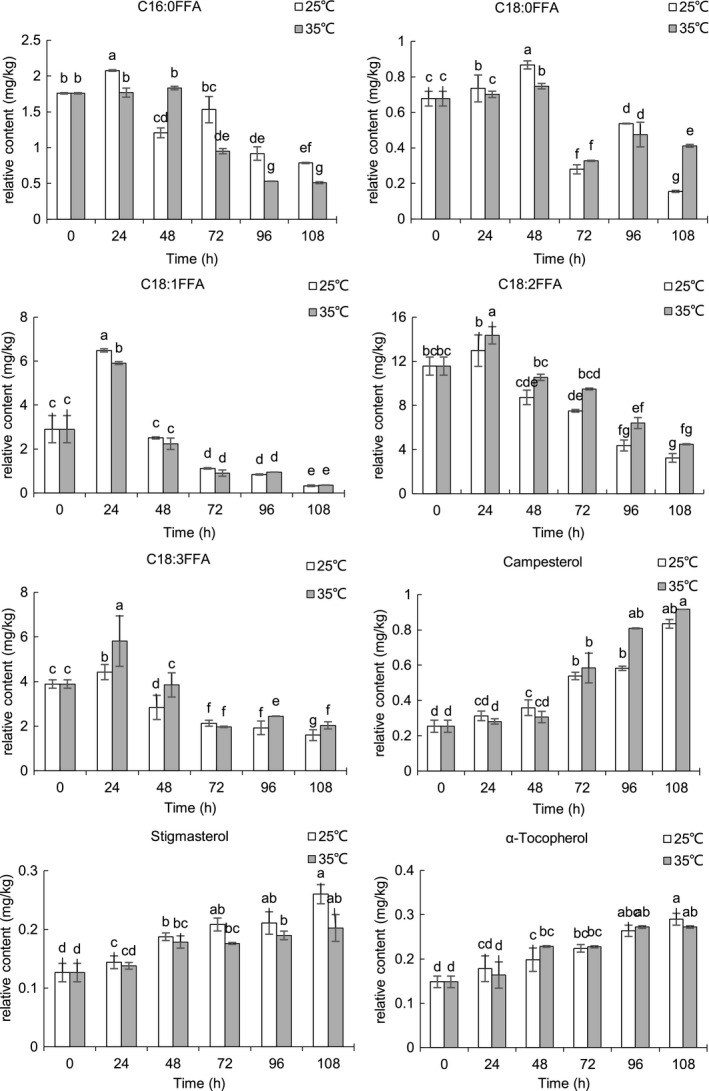
Relative content of representative compounds in fraction II of germinating sunflower seed

**FIGURE 6:**
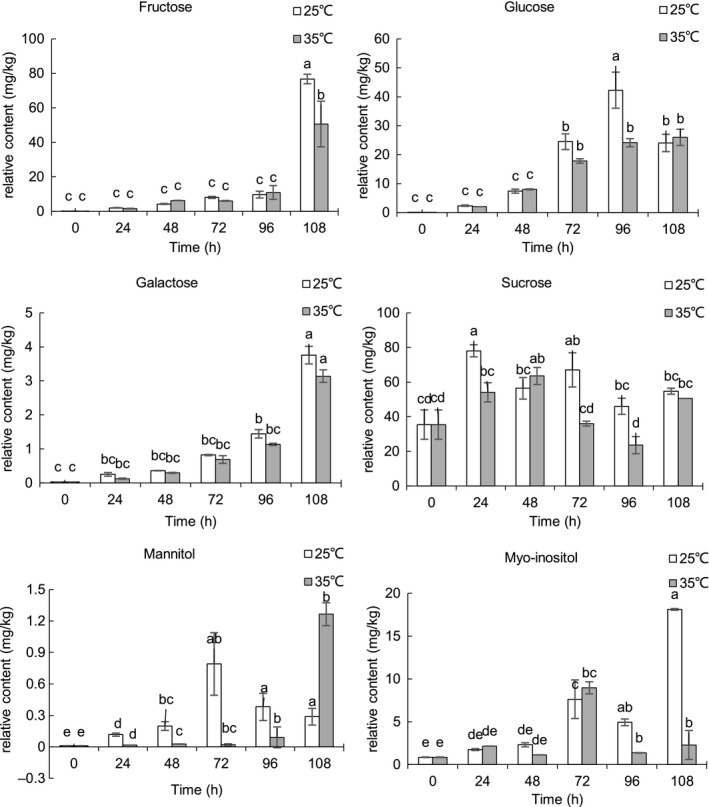
Relative content of representative compounds in fraction III of germinating sunflower seed

**FIGURE 7:**
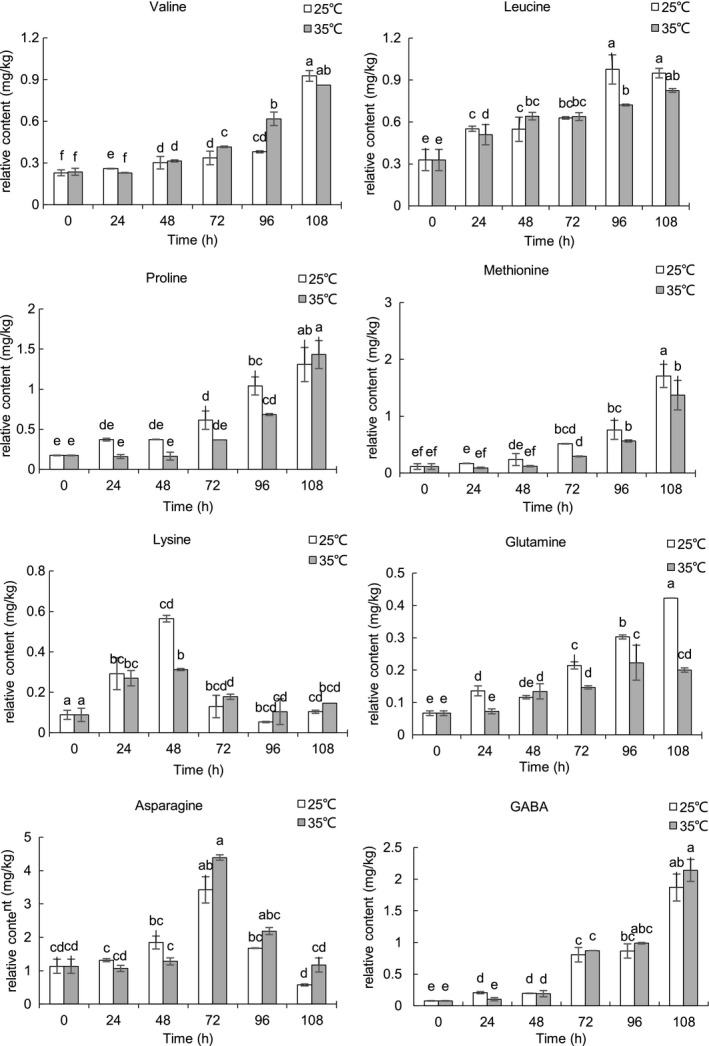
Relative content of representative compounds in fraction IV of germinating sunflower seed

The relative contents of the representative nonpolar and polar compounds of sunflower seeds during germinating were shown in Figures 4, 5, 6, 7. Triacylglycerols (TAGs) stored in the oil body are usually located near the glyoxysomes, which are organelles that convert fatty acids derived from TAGs into soluble‐sugar precursors through the glyoxylate cycle (Graham, 2008). Almost all TAGs present in oilseeds are lost during seed germination and seedling development (Kim et al., 2011; Rabiei et al., 2007; Tonguç et al., 2012). FAME in fraction I comes from transesterification of the lipid extract which reflects fatty acid compositions of the sunflower seed triglycerides. After 108 hr of germination, the content of FAME decreased during germination at 25 and 35°C, and the decrease of FAME content is an expected result in the process of embryo development as triglycerides are the main energy source of embryo development. Similar to our results, decreases in the content of FAME were observed in germinating rice(Shu et al., 2008). The content of C16:0, C18:0, C18:1, and C18:3 FAME was lower at most of the time point at 25°C compared with those at 35°C. The previous study reported that temperature has a significant effect on seed germination and seedling growth of sunflower, and the optimum temperature for germinating sunflower seed was about 25°C (Gay et al., 1991). There was no significant difference in the FAME of linoleic acid (C18:2) between the two temperatures. It is noted that the enzymes involved in lipid biosyntheses and germination conditions have a great influence on lipase, transacylase, and acyltransferase (i.e., temperature, moisture, and germination time). Lipase, known as hydrolase, can catalyze the hydrolysis of triglycerides to glycerol and fatty acids. Large molecules break down into smaller molecules by adding water in the hydrolysis reaction. It is reported that the optimum temperature for lipase is 24°C in germinating soybean (Gadge et al., 2011). The lower content of C16:0, C18:0, C18:1, and C18:3 FAME at 25°C may be possibly due to the higher lipase activity at 25°C. Squalene, the major representative of hydrocarbon and triterpene detected in fraction I, significantly decreased (*p* < .05) in the course of the germination. The content of squalene was significantly higher (*p* < .05) in sunflower seeds germinated at 25°C compared with sunflower seeds germinated 35°C. Squalene content was significantly (*p* < .05) higher at 25°C for 24 hr.

The dynamic time‐dependent changes of the representative compounds in fraction II were shown in Figure 5. The content of FFA was higher (except 18:0) at 24 hr than other times after that decreased significantly (*p* < .05) during germination. The increase of FFA content at the first 24 hr may be due to the mobilization of triacylglycerols that generates significant amounts of free fatty acids and glycerol (Bellieny‐Rabelo et al., 2016). FFA enters the glyoxysome for conversion to oxaloacetic acid, passes into the mitochondrion, and ultimately into the cytosol for conversion to sucrose, which then transported as an energy source from cotyledons to the growing axis of seedling(Graham, 2008). The increase of α‐galactosidase and lipase activities and decrease of lipoxygenase activities after germination shorter than 72 hr have been reported previously (Paucar‐Menacho et al., 2010). Higher germination temperatures from 25 to 32°C lead to lipoxygenase activities decrease(Paucar‐Menacho et al., 2010). The lower lipoxygenase activity at 25°C may be the reason for a higher content of C18:2 and C18:3 compared with that at 35°C. In contrast with our result, the small change in free fatty acids levels during germination was reported in the previous study (Marton et al., 2010).

Germination increased sterols content in sunflower seeds. The observation is consistent with the result of rice germination(Shu et al., 2008). A more pronounced increase of sterols was observed (on average, + 30%) in germinating soybeans after 120 hr of germination (Chandrasiri et al., 1990). At an initial germinating time (24 and 48 hr), the content of campesterol was significantly higher at 25°C, and after 48 hr, the content of campesterol increased significantly (*p* < .05) in sunflower seeds germinated at 25 and 35°C. There were no significant differences in the content of β‐sitosterol between 25 and 35°C during germination. The content of stigmasterol was significantly higher (*p* < .05) in sunflower seeds germinated at 25°C than that at 35°C. α‐Tocopherol content increased significantly (*p* < .05) with the increasing germination time at 25°C. At the initial (24 hr) and end of germination (108 hr), the content of α‐tocopherol in sunflower seeds germinated at 25°C was significantly higher (*p* < .05) than that at 35°C.

Free sugars detected in germinated sunflower seeds include monosaccharides ( glucose, fructose, and galactose), and disaccharides (sucrose) were shown in Figure 6, the content of soluble sugars (fructose, glucose, and galactose) remained low until their increase at 72 hr. Previous studies showed that a‐amylase activity continuously increased in germinating seeds (Choi et al., 1996), which may be the reason for the great increase of these soluble sugars during the late germination stage. The same results were reported by Ribeiro et al. (2015) that fructose levels sharply increased in germinating *Ricinus communis* seed. The other hydrolyzes probably contribute to the complete hydrolysis to monosaccharides and reducing sugars, for example, sucrose hydrolyze to glucose and fructose by invertase. The increase in reducing sugars was also reported by Moongngarm and Saetung (2010). The content of sucrose showed fluctuation during germination. In the process of seed germination and seedling development, the stored triacylglycerols are hydrolyzed by lipases on the main chain of glycerol in the oil body and converted into carbohydrates major as sucrose that may be led to an increase of sucrose at the initial stage of germination. The required energy is hypothesized to come from stored carbohydrates, sucrose, and raffinose family oligosaccharides (Peterbauer & Richter, 2001). In energy production, invertases are the main driving factor of sucrose utilization. Sucrose synthase can also catalyze the reversible decomposition of sucrose. These results support the increase of sucrose‐derived monosaccharides demand in the later stage of seed germination and the reason for the reduction of sucrose later in germination. There were no significant differences in the content of fructose, glucose, and galactose in sunflower seeds germinated at 25 and 35°C. The content of mannitol and myo‐inositol increased significantly (*p* < .05) in the course of germination. The content of mannitol and myo‐inositol was significantly higher (*p* < .05) in sunflower seeds germinated at 25°C compared with sunflower seeds germinated at 35°C. However, no consistent patterns were observed for glycerol and myo‐inositol in rice germination (Shu et al., 2008).

The relative contents of amino acids from sunflower seeds during germination were showed in Figure 7. Generally, the content of amino acids increased significantly (*p* < .05) after germination. The relative content of leucine, proline, methionine, glutamine, and GABA was higher at 25°C compared with those at 35°C. The increase of amino acids was the result of protein degradation by protease and new enzyme synthesis (Sibian et al., 2016). An inverse decrease in the total protein content of sunflower seed was observed throughout the germination period(Erbaş et al., 2016). The same results were derived by Moongngarm and Saetung (2010). The amino acid content of wheat was also higher after germination (Zörb et al., 2006). Proline accumulation is correlated with pyrroline‐5‐carboxylate synthetase (P5CS), which is the key regulatory and rate‐limiting enzyme in biosynthesis. Plants also synthesize proline from ornithine by ornithine aminotransferase (OAT) through intermediate pyrroline‐2‐carboxylate (Song et al., 2005). P5CS and OAT activities markedly declined with increasing temperature, and the activities are higher at 25°C (Song et al., 2005). The activation of several amino acid biosynthesis pathways in the germination process was reported by Bellieny‐Rabelo et al. (2016). A key step in aromatic amino acid biosynthesis is the production of chorismate via Shikimate pathway, which starts with the rate‐limiting reaction catalyzed by 3‐deoxy‐7‐phosphoheptulonate (DAHP) synthase. The amino acids produced by protein reserve hydrolysis can not only be used to synthesize new components, but also be the source of energy (Chen et al., 1975). Some amino acids may decompose more easily than others, which is another potential source of protein pattern during germination (Rodríguez et al., 2008). Lysine firstly increased and sharply decreased at 72 hr (*p* < .05) during germination. The decarboxylation of lysine results in the formation of cadaverine which is mainly concentrated on the embryonic axis (Shalaby, 2000; Torrigiani & Scoccianti, 1995). An inverse linear correlation between the lysine and nonprotein nitrogen was observed in germinating legumes (Rodríguez et al., 2008). Compared with raw sunflower seeds, glutamic acid content increased during the germination of sunflower seeds and increased more at 25°C for 24 and 48 hr. while reached the maximum value at the final stage of germination at 35°C. GABA, a well‐known biogenic amine, is directly responsible for regulating muscle tension in humans (Liu et al., 2016). At 108 hr, the content of GABA exhibited a tenfold increase in germinating sunflower seeds compared with the raw seeds. Under different temperature, the content of GABA was also different (Xu & Hu, 2014). The GABA content was higher in sunflower seeds germinated for 24 hr at 25°C, but lower at 96 and 108 hr compared with that at 35°C. The temperature and time of germination significantly affect the content of GABA, and related enzyme activities (Xu & Hu, 2014). The glutamic acid and GABA content and GABA transaminase (GABA‐T) and glutamate decarboxylase (GAD) activities showed a positive correlation. GAD is the rate‐limiting enzyme of GABA synthesis. When the substrate glutamic acid reaching the recommended level for GAD reactions, GABA of soybean increased with the increase of GAD activity (Oh & Choi, 2001), and a similar result was observed in brown rice (Liu et al., 2005). The temperature has a great influence on GAD activity which was increased with the temperature from 19 to 32°C at the same germination stage. The best temperature for improving GAD activity and accumulating GABA was 30°C (Zhang et al., 2007). The increase of GABA content may be related to the increase of GAD activity and glutamic acid. The increase of GABA content during germination has also been reported in Bariey (Frank et al., 2011) and rice (Shu et al., 2008). Glutamine increased with germination time. The content was higher at 25°C compared with that at 35°C. Glutamine synthetase activity was increased with the germination of the seed until the cotyledon to begin green and then was reduced gradually. Asparagine increased firstly and then decreased quickly thereafter, which was lower than that of raw sunflower seeds. The results of enzymolysis of reserve protein demonstrated that 84% of these aspartyl residues could be used as asparagine(Atkins et al., 1975); thus, asparagine content increased in the early germination, and then, demand for protein synthesis established an increasingly large asparagine library that led to the reduction of asparagine.

## 4 CONCLUSIONS

The data obtained demonstrated the applicability of the metabolomics approach in tracking the metabolites changes during sunflower seed germination. The combination of extraction and fraction method can cover a broad spectrum of polar and nonpolar compounds, including not only representatives of primary plant metabolism but also metabolites related to nutrition. PCA results showed that the metabolite changes during germination were reflected by the time‐dependent shifts of scores. Agglomerative hierarchical clustering revealed the samples could be divided into three clusters based on the similarity between each pair of objects: without germination, early germination phase, and late germination phase. Relative content using heatmap of the metabolites showed decreases in the content of FAME and FFA, and increases in the content of soluble sugar, sterol, and amino acid during seed germination. Based on the metabolite changes, 25°C was better for the accumulation of α‐tocopherol, stigmasterol, and amino acids. The best germinating temperature was 25°C.

## CONFLICT OF INTEREST

The authors have declared no conflicts of interest in this article.
